# Isolation and Characterization of CsWRKY7, a Subgroup IId WRKY Transcription Factor from *Camellia* *sinensis*, Linked to Development in Arabidopsis

**DOI:** 10.3390/ijms20112815

**Published:** 2019-06-09

**Authors:** Wei Chen, Wan-Jun Hao, Yan-Xia Xu, Chao Zheng, De-Jiang Ni, Ming-Zhe Yao, Liang Chen

**Affiliations:** 1 Key Laboratory of Tea Biology and Resources Utilization, Ministry of Agriculture; Tea Research Institute Chinese Academy of Agricultural Sciences, 9 South Meiling Road, Hangzhou 310008, Zhejiang, China; chenwei2551@163.com (W.C.); haowanjun@tricaas.com (W.-J.H.); xuyanxia@tricaas.com (Y.-X.X.); zhengchaotea@163.com (C.Z.); 2 College of Horticulture and Forestry Science, Huazhong Agricultural University, 1 Shizishan Street, Hongshan District, Wuhan 430070, Hubei, China; nidj@mail.hzau.edu.cn

**Keywords:** subgroup IId, CsWRKY7, flowering, Arabidopsis, *Camellia sinensis*

## Abstract

WRKY transcription factors (TFs) containing one or two WRKY domains are a class of plant TFs that respond to diverse abiotic stresses and are associated with developmental processes. However, little has been known about the function of *WRKY* gene in tea plant. In this study, a subgroup IId *WRKY* gene *CsWRKY7* was isolated from *Camellia sinensis*, which displayed amino acid sequence homology with Arabidopsis *AtWRKY7* and *AtWRKY15*. Subcellular localization prediction indicated that CsWRKY7 localized to nucleus. Cis-acting elements detected in the promotor region of *CsWRKY7* are mainly involved in plant response to environmental stress and growth. Consistently, expression analysis showed that *CsWRKY7* transcripts responded to NaCl, mannitol, PEG, and diverse hormones treatments. Additionally, *CsWRKY7* exhibited a higher accumulation both in old leaves and roots compared to bud. Seed germination and root growth assay indicated that overexpressed *CsWRKY7* in transgenic Arabidopsis was not sensitive to NaCl, mannitol, PEG, and low concentration of ABA treatments. *CsWRKY7* overexpressing Arabidopsis showed a late-flowering phenotype under normal conditions compared to wild type. Furthermore, gene expression analysis showed that the transcription levels of the flowering time integrator gene *FLOWERING LOCUS T* (*FT*) and the floral meristem identity genes *APETALA1* (*AP1*) and *LEAFY* (*LFY*) were lower in *WRKY7*-OE than in the WT. Taken together, these findings indicate that CsWRKY7 TF may participate in plant growth. This study provides a potential strategy to breed late-blooming tea cultivar.

## 1. Introduction

Plants suffering from diverse abiotic stresses in the developmental process have evolved and obtained a series of mechanisms to combat with these environmental stresses. Majority of transcription factors (TFs) participate in these adaptive mechanisms [1]. WRKY TFs—a large family of transcription factors in plants—are involved in response to multiple stresses and external stimuli [2,3,4].

WRKY transcription factor is named after the WRKY domain, which contains one or two highly conserved WRKYGQK motifs and one zinc-finger motif [5,6]. According to the number of WRKY domains and the type of zinc finger motif, WRKY TFs can be divided into three types: Group I, containing two WRKY domains and one C2H2 zinc-finger structure. Group II, containing one WRKY domain, and sharing the same zinc finger structure with group I. In addition, based on the amino acid sequence of the DNA-binding domain, Group II can be further divided into IIa, IIb, IIc, IId, and IIe subgroups; Group III also contains one WRKY domain, but its zinc finger structure is C2HC [4,7].

SWEET POTATO FACTOR1 (SPF1) specifically binding to W-box, the first defined WRKY TF, negatively regulates the expression of storage proteins and β-amylase in sweet potato [8]. Since then, considerable efforts have been made to unveil the role of WRKY TFs in plant response to biotic and abiotic stresses. In *Arabidopsis thaliana*, it has been demonstrated that WRKY TFs contribute to pathogen resistance, salinity, heat, and drought stress responses [7,9,10,11]. W-box (TTGACT/C) was present in the promoters of several stress-associated genes. Since WRKY proteins bind to W-box, they induce the expressions of these stress-associated genes [4,5,11,12,13]. Many studies have shown that WRKY proteins participate in secondary metabolism (phenylpropane, alkaloids, and terpenes.), and hormone signaling [10]. Moreover, WRKY proteins have been demonstrated to involve in plant growth processes, such as leaf senescence, shoot branching, trichome, and flowering. For instance, potato ScWRKY1 [14], Arabidopsis TTG2 [15], and MINISEED3 are involved in seed growth [16]; Arabidopsis AtWRKY6, -45, -53, -57, and -70 are involved in senescence regulation [17,18,19,20,21]; Arabidopsis AtWRKY12, -13, -75, -34, mango MIWRKY12, soybean GsWRKY20, and rice DIf1 have been reported to be involved in plant flowering regulation or pollen development [22,23,24,25,26,27]. However, few studies have reported WRKY TFs in tea plant.

Tea plant (*Camellia sinensis* L.) is an important commercial woody crop. As a nonalcoholic beverage, tea is processed from tea leaves and is widely consumed worldwide. Early blooming in tea plant and adverse environmental factors affect the quality and yields of tea. Increasing studies have demonstrated that WRKY TFs play pivotal roles in plant growth and abiotic stresses. In one previous study, two WRKY TFs—CsWRKY31 and CsWRKY48—were reported to participate in O-methylated catechin biosynthesis in tea plant (*Camellia sinensis*) [28]. Several *CsWRKY* genes were induced by diverse stresses such as temperature, ABA, and NaCl [29,30,31]. However, the roles of CsWRKYs in plant growth and development remain unclear. The current study aims to provide a functional characterization of *CsWRKY7*, a member of the group IId WRKY family in tea plant. CsWRKY7 was a close homolog of Arabidopsis *AtWRKY7* and *AtWRKY15*, two well-characterized Group IId WRKY proteins with important roles in plant defense, leaf senescence, and abiotic stress responses [32,33,34]. When it is exposed to diverse stresses, *CsWRKY7* is upregulated and localized to the nuclei in both tobacco leaves and Arabidopsis roots. *CsWRKY7*-overexpressing Arabidopsis did not respond to abiotic stresses. However, the overexpression of *CsWRKY7* delayed flowering. Gene analysis revealed the downregulation of several flowering-related genes in *CsWRKY7* overexpression lines. A better understanding of the flowering mechanisms is conducive to breeding late-blooming tea plants.

## 2. Results

### 2.1. Isolation and Characterization of CsWRKY7 from C. sinensis

**Figure 1:**
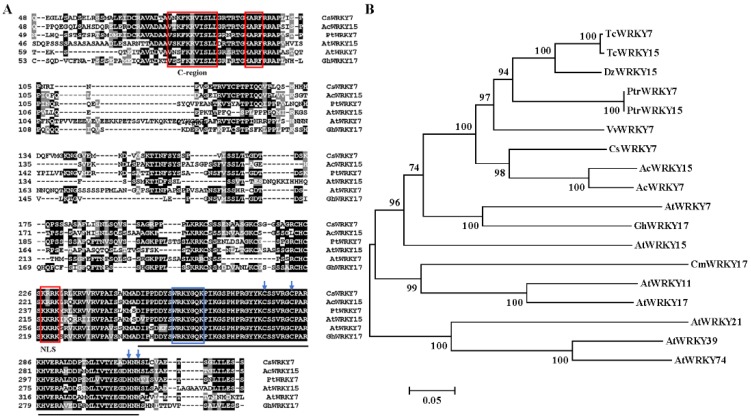
Protein sequence alignment and phylogenetic relationship of CsWRKY7 (**A**) Sequence alignment of the deduced CsWRKY7 protein with other Group IId WRKY homologs. Black lines highlight the conserved region of WRKY. Blue box and arrows highlight the WRKY domain and the zinc-finger motif, respectively. The conserved primary sequences—HARF motif, C-region, and putative NLS—are boxed in red. (**B**) The phylogenetic tree of CsWRKY7 and 17 other group IId WRKY subfamily members. Accession number for group IId WRKY members: AtWRKY7 (AAK28440), AtWRKY11 (AAK96194), AtWRKY15 (AF224704), AtWRKY17 (AAL13049), AtWRKY21 (AAK28441), AtWRKY39 (AAK96198), AtWRKY74 (AAL35291), AcWRKY7 (PSS36058.1), AcWRKY15 (PSS21265.1), VvWRKY7 (RVX23377.1), PtrWRKY7 (XP_006380693.1), PtrWRKY15 (XP-002310122), GhWRKY17 (HQ651068), TcWRKY7 (EOX91521), TcWRKY15 (XP-007047365), CmWRKY17 (AJF11725), and DzWRKY15 (XP-022740807).

*CsWRKY7*, one *WRKY* gene, was amplified from tea leaves cDNA by RT-PCR. This amplified *CsWRKY7* contained the complete open reading frame (ORF) of 972 bp encoding 323 amino acids. CsWRKY7 protein had a predicted molecular mass of 35.37 kDa and an isoelectric point of 9.47. Sequence analysis showed that CsWRKY7 in tea plant shared a high similarity (71.94%) to AcWRKY15 in kiwifruit (Genbank: PSS21265). Additionally, CsWRKY7 was found to have a nuclear localization signal (NLS) at 227–230 amino acid region, and have two motifs, namely, HARF structure and a shorter conserved structural motif (C-region which is known as a Ca^2+^-dependent calmodulin-binding domain) (Figure 1A). Thus, CsWRKY7 was assigned to group IId subfamily. Phylogenetic analysis showed that CsWRKY7 was closely related to AcWRKY15, PtrWRKY7, VvWRKY7, AtWRKY7, and AtWRKY15 (Figure 1B). AtWRKY7 and AtWRKY15 TFs have been reported to be involved in plant defense response to bacterial pathogens, leaf senescence, or mitochondrial-mediated osmotic stress [32,33,34], their homologous genes *CsWRKY7* was predicted to be a TF that may participate in plant development and respond to stress treatment.

### 2.2. Sequence Analysis of CsWRKY7 Promoter

According to ‘Shuchazao’ tea genome data, the promoter sequence of *CsWRKY7* was amplified by PCR. A 1680 bp *CsWRKY7* promoter sequence was cloned and the putative cis-elements were predicted through the PlantCARE database. Two functional elements—TATA-box and CAAT-box—were widely distributed in the promoter region. Additionally, a group of elements which respond to such environmental stresses as phytohormone stress (salicylic acid and auxin), light, plant growth (pollen), and abiotic stresses (anaerobic, sugar, wounding, NaCl, dehydration, and heat) were found (Table 1). Interestingly, many MYB-recognition sites were present in the promoter region of *CsWRKY7*, and these motifs also existed in dehydration-responsive gene *RD22A*, indicating that *CsWRKY7* might be involved in dehydration stress and be modulated by MYB members. In addition, *CsWRKY7* promoter region existed two W-boxes, which specially bind to WRKY TFs. These prediction results indicated that CsWRKY7 TF may play a vital role in stress responses and plant growth through multiple signal transduction pathways.

### 2.3. Subcellular Localization of CsWRKY7

**Figure 2:**
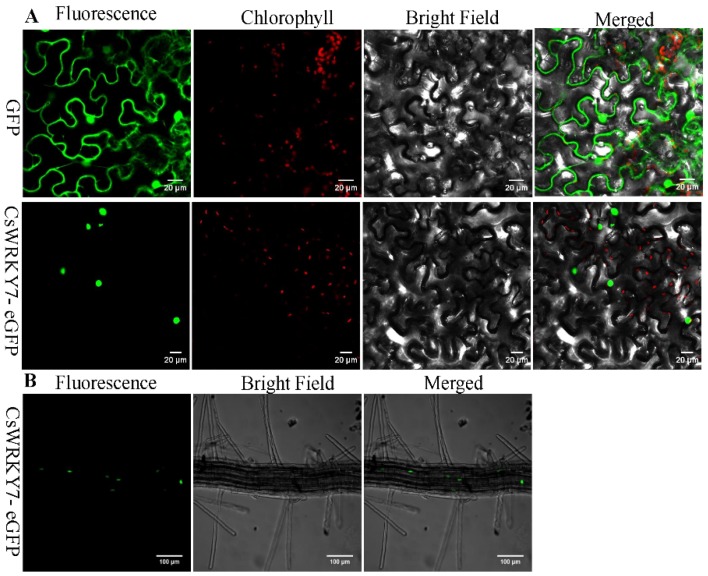
Subcellular localization analysis of CsWRKY7 protein: (**A**) GFP alone (upper panel) and *CsWRKY7*-eGFP (middle panel) were transiently expressed in tobacco epidermal cells. Representative images from left to right in each panel were taken under fluorescence, chlorophyll, transmitted light and an overlay of both channels. Scale bar = 20 μm. (**B**) The roots of *CsWRKY7*-eGFP (bottom panel) in transgenic Arabidopsis was used for observation of GFP fluorescence. Representative images from left to right were taken under fluorescence, bright field and an overlay of both channels. Scale bar = 100 μm.

To examine the localization of CsWRKY7, the full-length cDNA of *CsWRKY7* was fused to enhanced green fluorescent protein (eGFP). As shown in Figure 2A, *CsWRKY7*-eGEP was localized to the nucleus when it was transiently expressed in *Nicotiana benthamiana* leaves. The green fluorescence was observed both in the cell membrane and nucleus in the GFP control vector. Similarly, green fluorescence was also observed in the nucleus of transgenic Arabidopsis seedlings (Figure 2B). These results showed that CsWRKY7 was targeted to the nucleus.

**Figure 3:**
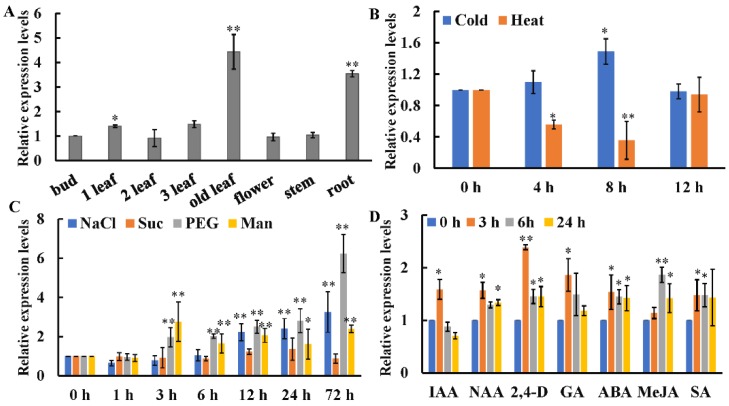
Relative expression levels of *CsWRKY7* in tea plants (**A**) Tissue expression profiles of *CsWRKY7* in ‘Longjing 43’. Different tissues include bud, 1st leaf, 2nd leaf, 3rd leaf, old leaf, flower, stem, and root. The expression levels of *CsWRKY7* in different tissues were compared with the bud. (**B**) The transcript levels of *CsWRKY7* under cold (4 °C) and heat (38 °C). *CsWRKY7* expression levels are detected at four different time points (0, 4, 8 and 12 h) post-temperature stress treatment. (**C**) Relative expression levels of *CsWRKY7* under different abiotic stress. Two-year-old tea seedlings were treated with 150 mM NaCl, 90 mM sucrose (Suc), 10% (*w*/*v*) PEG4000 (PEG) and 90 mM mannitol (Man), and samples were harvested at the time intervals indicated. (**D**) The transcript levels of *CsWRKY7* under various phytohormone, including 100 μM indolyl-3-acetic acid (IAA), 5 μM naphthalene-1-acetic acid (NAA), 5 μM 2,4-dichlorophenoxyacetic acid (2,4-D), 100 μM abscisic acid (ABA), 50 μM methyl jasmonate (MeJA), 5 mM salicylic acid (SA), and 100 μM gibberellins (GA), which were added to the culture solution, the functional leaves were harvested at 0, 3, 6, or 24 h post-treatment. Treated samples at 0 h served as controls. Error bars represent ± S.E. for three independent experiments. The significant level is presented by the asterisks (* *p* < 0.05, ** *p* < 0.01).

The qRT-PCR result indicated that *CsWRKY7* was expressed in all detected tissues. The expression level in old leaves was approximately 4.4 times as high as that in buds (Figure 3A). To investigate the role of CsWRKY7 TF in abiotic stresses, we analyzed the expression patterns of *CsWRKY7*, when it was exposed to temperature, sodium chloride (NaCl), sucrose (Suc), polyethylene glycol (PEG), and mannitol (Man) stresses. As shown in Figure 3B,C, in leaves, *CsWRKY7* was downregulated under high temperature stress, especially at 8 h, its expression level decreased by 65%. When exposed to low temperature, the expression level of *CsWRKY7* was not significantly changed at 4 h or 12 h. However, the expression level of *CsWRKY7* was upregulated under the treatments of NaCl, Man, and PEG. For example, its expression level increased gradually after NaCl and PEG treatment for 3 h, reaching a peak of 3.26- and 6.23- fold at 72 h, respectively. Though two sugar-responsive elements existed in the promoter region, no significant difference in the expression level of *CsWRKY7* after sucrose treatment. In order to investigate whether *CsWRKY7* expression was regulated by phytohormones, two-year-old tea plants were exposed to the major plant hormones including indolyl-3-acetic acid (IAA), naphthalene-1-acetic acid (NAA), 2,4-dichlorophenoxyacetic acid (2,4-D), abscisic acid (ABA), methyl jasmonate (MeJA), salicylic acid (SA), and gibberellins (GA). As presented in Figure 3D, the expression level of *CsWRKY7* was elevated under these hormone treatments except IAA. These results implied that *CsWRKY7* might be involved in the regulation of abiotic stress and hormones networks.

### 2.4. Overexpression of CsWRKY7 Affects Flowering in Transgenic Arabidopsis

Seed Germination and Root Growth in Transgenic Plants under Abiotic Stresses

**Figure 4:**
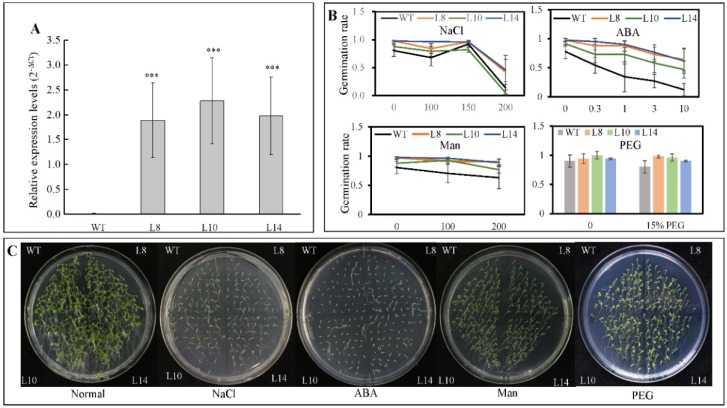
Germination rates of *CsWRKY7*-overexpressing Arabidopsis lines under different stress conditions (**A**) Expression of *CsWRKY7* in the leaves of WT and three transgenic lines (L8, L10, and L14), respectively. Data are shown as the mean ± S.E. (*n* = 3). (**B**) The germination rate of WT and transgenic lines. Their seeds grown on the 1/2 MS supplied with different concentrations of NaCl, ABA, mannitol and PEG for 4 days. Experiments were performed in five biological replicates. Fifty seeds of each WT and three transgenic lines were germinated in one replicate. Data are shown as the mean ± S.E. (**C**) Germination performance of WT and transgenic lines were taken under normal conditions, 150 mM NaCl, 0.3 μM ABA, 200 mM mannitol, and 15% PEG treatment for 7 days. Asterisk indicated that the expression level is significantly different from the value of the control (‘***’ *p* < 0.001).

To assess the function of CsWRKY7 TF, an expression construct pH7FGW2.0-*CsWRKY7* was transformed into *A. thaliana*. Three homozygous transgenic lines—L8, L10, and L14—were confirmed by real-time PCR with *Actin-2* gene serving as an internal reference. As shown in Figure 4A, the *CsWRKY7* transcript levels were significantly higher in transgenic lines than in wild type.

**Figure 5:**
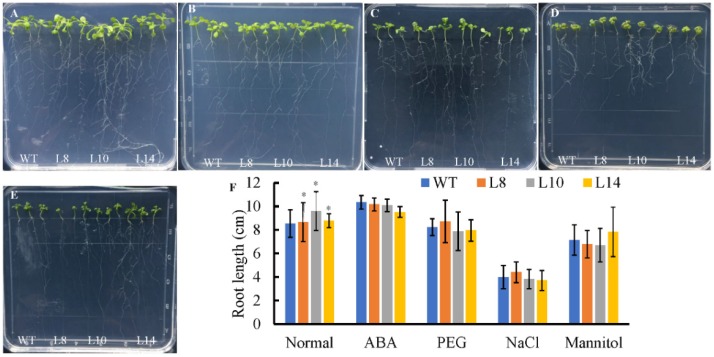
Root growth of *CsWRKY7*-overexpressing Arabidopsis lines under different stress conditions. Seeds were germinated for 4 days on 1/2 MS medium, and the seedlings were then transferred to 1/2 MS medium with or without different treatment for 10 days. (**A**) Normal condition, (**B**) 150 mM NaCl treatment, (**C**) 0.3 μM ABA treatment, (**D**) 200 mM mannitol treatment, and (**E**) 15%PEG6000 treatment, and (**F**) root length were measured at 10 d after the transfer, each line included three seedlings, experiments were performed in four biological replicates. Data are represented as the mean ± SE of 12 seedlings. The significant level is presented by the asterisks (* *p* < 0.05).

Since *CsWRKY7* responds to abiotic stress and hormone treatments, seed germination was tested with WT and homozygous 35S::*CsWRKY7* transgenic lines to determine the specific role of CsWRKY7 TF in abiotic stress. Though the germination rate of overexpressing lines was higher than that of WT (Figure 4B,C), the difference was not statistically significant under normal growth condition or under abiotic stresses, suggesting that *CsWRKY7* overexpressing lines may not be sensitive to the above induction during seed germination. No significant difference in root growth was observed between WT and overexpressing lines in the presence of different stress media (Figure 5). However, the root elongation of 35S::*CsWRKY7* lines were higher than that of WT with 1/2 MS medium, indicating that CsWRKY7 might promote root growth at the seedling stage under normal growth condition.

### 2.5. CsWRKY7 Overexpressing Lines Exhibit the Phenotype of Delayed Flowering

**Figure 6:**
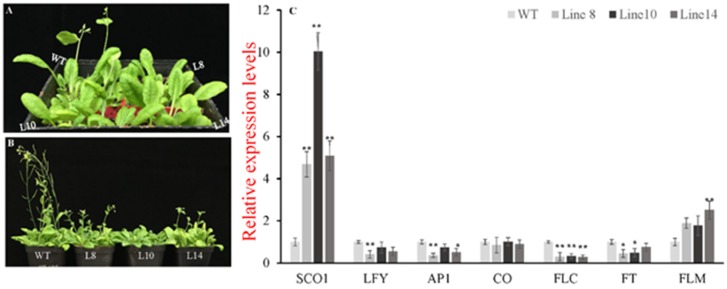
Identification of the *CsWRKY7* in transgenic Arabidopsis. (**A**,**B**) Overexpression of *CsWRKY7* delayed Arabidopsis flowering in different developmental stages: (**A**) Representative photographs of 25-day-old-plant of WT and transgenic lines (L8, L10, and L14) growing in normal conditions. (**B**) 35-day-old-plant. Each line included four seedlings. Experiments were performed in five biological replicates. (**C**) Expression patterns of flowering-related genes in *CsWRKY7*-overexpressing and wild type Arabidopsis. Leaf samples were harvested from 25-day-old transgenic lines and WT. The error bars indicate the means ± S.E. (*n* = 5), * indicates that the differences are significant (*p* < 0.05), ** indicate that the differences are highly significant (*p* < 0.01).

In order to investigate whether CsWRKY7 TF participated in plant growth and development, the phenotype of *CsWRKY7*-overexpressing lines and WT was observed during plant growth process. As shown in Figure 6A, B, WT plants bolt earlier than transgenic lines after 25-day growth. When wide-type plants were in silique stage, *CsWRKY7* overexpressing plants were in bolting stage or vegetative growth stage after 35-day growth. To elucidate the regulation mechanism of *CsWRKY7* in flowering, several flowering-related genes was analyzed (Figure 6C). *SUPPRESSOR OF CONSTANS 1* (*SCO1*), a floral integrator, was significantly induced in 35S::*CsWRKY7* lines compared to WT. No noticeable difference in the relative expression level of *CONSTANS* (*CO*) gene that the central regulators in the photoperiod pathway was observed between WT and transgenic lines. As a long-distance transport signal, *FLOWERING LOCUS T* (*FT*) was significantly downregulated in transgenic lines. Simultaneously, two important genes related to inflorescence meristem—*APETALA1* (*AP1*) and LEAFY (*LFY*)—were significantly suppressed in Line 8. The expression levels of *AP1* and *LFY* were downregulated by 64% and 60%, respectively. However, a flowering inhibitor, *FLOWERING LOCUS C (FLC)*, was significantly downregulated in overexpressing lines, while its homologous gene *FLOWERING LOCUS M (FLM)* was upregulated in 35S::*CsWRKY7* lines, compared to WT. Therefore, we speculate that CsWRKY7 delay flowering time might through inhibiting the transcription level of *AP1* and *LFY*.

## 3. Discussion

WRKY TFs widely participate in plant stress responses and plant growth and development. This study indicated that *CsWRKY7* is involved in abiotic stresses. Constitutive overexpression of *CsWRKY7* not only promotes root growth, but also delays the flowering time in transgenic Arabidopsis plants by suppressing flowering-related genes.

CsWRKY7, homologous to AtWRKY7 and AtWRKY15, belongs to subgroup IId of WRKY family. AtWRKY7 transcription factor not only acted as a negative regulator in PAMP-mediated immune response but also participated in leaf senescence [34,35,36]. *CsWRKY7* was abundant in old leaves and roots in tea plant, which was somewhat similar to the expression patterns of *AtWRKY7* in Arabidopsis leaves, thus it could be speculated that CsWRKY7 might be involved in tea growth and development. Besides, *CsWRKY7* gene expression was induced by various osmotic stresses and hormones exposure (Figure 3). Sequence analysis identified several potential stress-responsive elements in the promoter region including WBOXHVISO1, CCAATBOX1, GT1GMSCAM4, and MYB1AT and pollen-specific cis-regulatory elements (GTGANTG10), which participated in regulating the response to sugar, heat, NaCl, and drought stresses, respectively (Table 1). Some previous studies have reported that these cis-regulatory elements were stress-related. For example, MYB1AT existed in the promoter region of the Arabidopsis dehydration gene *RD22*, and GT1GMSCAM4 was present in the promoter region of the pathogen and salt-inducible gene *SCaM-4*, which consisted with the cis-regulatory elements in the promoter of *CsWRKY7*, suggesting that *CsWRKY7* participated in various stress responses. (Figure 3) [37,38]. Though two pollen-specific cis-elements present in the promoter, the expression level of *CsWRKY7* in flower was not significantly different from that in bud, which might be probably due to the complicated regulation mechanisms of gene expression. Besides, two hormone response elements—SA and auxin-response elements—are also found in this region. The hormone induction experiment showed that *CsWRKY7* gene was responded to gibberellin and auxin treatment (NAA and 2,4-D), indicating that CsWRKY7 might also be involved in GA- and auxin- and abiotic stress-mediated signaling, but the corresponding mechanism remains be further explored.

Plant growth such as flowering, is a sophisticated regulated process that can be affected by diverse environmental stimuli. Previous research has reported that several WRKY members participate widely in plant growth and development. AtWRKY44 was reported to be involved in trichome and seed coat development in Arabidopsis [15]. AtWRKY4/-6/-7/-11/-57 were involved in leaf senescence [4]. WRKY transcription factors were involved in the regulation of plants flowering. For example, AtWRKY75 directly bound to the promoter region of *FT* gene, thereby positively regulating Arabidopsis flowering [27]. Both AtWRKY12 and AtWRKY13 transcription factors regulated Arabidopsis flowering time under short day [26]. Additionally, mango MlWRKY12 [24], soybean GsWRKY20 [23], and rice OsWRKY11 [25] also had a regulatory role in flowering. However, few studies of WRKY TFs in tea plant were reported, especially their functions related to plant development. In this study, its ORF was overexpressed in Arabidopsis to determine whether or not CsWRKY7 involved in plant growth. Transgenic analysis indicated that the overexpression of CsWRKY7 altered growth and flowering time of transgenic Arabidopsis (Figure 5 and Figure 6). Gene analysis showed that two meristem identity genes including *AP1* and *LFY* were downregulated in transgenic Arabidopsis, compared with WT (Figure 6). Interestingly, previous studies have reported that there existed one or more W-boxes in the promoter region of *AP1* and *LFY* [39]. As noted earlier, WRKY TFs could specifically bind to W-box [4,5,11,12,13]. Therefore, the reason why *CsWRKY7* overexpressing lines delayed flowering may lie in that *CsWRKY7* gene directly bound to the promoter regions of *AP1* and *LFY* and inhibited their transcription levels.

However, *FLC,* a suppressor in flowering, was significantly downregulated in transgenic lines, while its homologous gene *FLM* was upregulated, indicating that *FLM* and *FLC* regulated flowering through different pathways. This finding was consistent with that of Katia (2003) [40]. *CO* gene encoding a B-box protein activated the transcription of *FT*. This study indicated that no difference in the expression level of *CO* was observed between transgenic lines and wild type, whereas the downstream gene *FT* was significantly inhibited, indicating that *FT* gene might be inhibited by other complexes such as PRC2, LHP1, SMZ, and TEM [41,42]. Hence, it could be speculated that *CsWRKY7* gene may be involved in flowering regulation independent of the autonomous and vernalization pathway. Generally, these data support our hypothesis that *CsWRKY7* delays flowering. The potential role of *CsWRKY7* as a negative regulator in flowering sheds new light on the development of tea plants at transcription level, but its regulation mechanism should be further explored.

Although *CsWRKY7* was induced by some abiotic stresses in *C. sinensis*, no significant difference in seed germination rate and seedling root growth was observed between *CsWRKY7* overexpressing Arabidopsis lines and WT under abiotic stresses (salt, mannitol, PEG, and exogenous ABA) (Figure 4 and Figure 5), which might be possibly due to the lack of correlation between the levels of mRNA and protein encoded by CsWRKY7, or due to the effect of the inserted transgene on the phenotype. However, the function of *CsWRKY7* gene in terms of osmotic stress response remains to be further characterized. The results of our study not only reveal an important role of CsWRKY7 in plant development, but also provide a foundation for breeding late-blooming tea plants.

To summarize, this study determined the response of *CsWRKY7* to various abiotic stresses and hormones treatments. Our data revealed that CsWRKY7 delayed flowering and promoted root growth in Arabidopsis. Nevertheless, it is necessary to investigate further the pathways through which CsWRKY7 regulates both the development and stress response of tea plant.

## 4. Materials and Methods

### 4.1. Plant Materials, Growth Conditions, and Stress Treatments

Two-year-old tea seedlings (*Camellia sinensis* cv. ‘Longjing 43′) were grown in the greenhouse of Tea Research Institute of the Chinese Academy of Agricultural Sciences (TRICAAS).

The buds, tender stem, flowers, and roots, and leaves at different developmental stages were collected for tissue-specific analysis. The methods of abiotic stresses were essentially the same with those described in our previous research [43] and the materials were collected for further analysis. *N. benthamiana* was used for protein subcellular localization, and was grown in a climate chamber (24 ± 2 °C, 70% relative humidity, and 12 h/12 h light–dark photoperiod). *A. thaliana* ecotype, Columbia-0 (Col-0), was used as the background material and experiment control. The seeds of wild type and transgenic lines were surface sterilized with 75% ethanol and 0.01% (*v*/*v*) Tween-20 for 8–10 min, and then were washed by distilled water 3−4 times. The seeds were placed on 1/2 MS medium plate at 4 °C in darkness for 48 h, then grown in a climate incubator under a 16 h day/8 h night cycle, respectively at 22 °C/20 °C.

### 4.2. Cloning of CsWRKY7 Gene and Sequence Analysis

The expressed sequence tags (EST) were obtained from different transcriptome databases [44,45,46]. The full length of CsWRKY7 cDNA sequence was identified from different tissues of ‘Longjing 43′ by KOD DNA Polymerase (Toyobo, Tokyo, Japan) and RT-PCR. Gene-specific primers were as follows; 5′-ATGGCCGTCGAGCTCGTGAT-3′ (forward) and 5′-TCAAGAAGACTCTAAGATAAG-3′ (reverse). The purified RT-PCR products were inserted into pEASY-blunt simple cloning vector (TransGen Biotech, Beijing, China) and subsequently sequenced. The predicted molecular weight and theoretical isoelectric point were analyzed by ProtParam (http://web.expasy.org/protparam/, accessed on: 22 May 2017) [47]. The homology of CsWRKY7 protein with other species was analyzed by NCBI BLAST website. The amino acid sequences of WRKY II d subfamily members were analyzed by ClustalX 2.0 and DNAMAN 6.0 software. The MEGA 5.0 software was used to analyze their evolutionary relationships. CsWRKY7 genomic DNA (gDNA) sequence was searched in ‘Shuchazao’ tea plant genome database, and the promoter region was determined by aligning the open reading frames of the gDNA sequence and those of corresponding gene. The promoter region of CsWRKY7 (1680 bp upstream of start codon) was amplified from ‘Longjing 43′ gDNA by PCR using KOD DNA polymerase (Toyobo). The regulatory elements in promoter region were predicted by PlantCARE database (http://bioinformatics.psb.ugent.be/webtools/plantcare/html/, accessed on: 9 March 2019) [48].

### 4.3. Expression Patterns of CsWRKY7 in Tea Plant

Total RNA was extracted from the different tissues of ‘Longjing 43′ using QIAGEN RNeasy Mini Kit (Qiagen, Hilden, Germany). The reverse transcription reaction was carried out by FastKing gDNA Dispelling RT SuperMix RT Reagent Kit (TIANGEN, Beijing, China). Real-time PCR was performed on an optical 384-well plate with a LightCycler 480 machine (Roche, Sussex, UK). Each reaction contained 5 μL of SYBR Green I Master Mix (Roche Diagnostics), 1.0 μL cDNA samples, and 0.4 μM of each gene specific primer in a final volume of 10 μL. The *glyceraldehyde-3-phosphate dehydrogenase* (*GAPDH*) gene (accession no. FS952640) was used as an internal control. The expression levels were computed by the formula 2^−ΔΔCt^ [49,50].

### 4.4. Subcellular Location Analysis

Using gateway cloning technology, *CsWRKY7* full-length cDNA sequence without terminator codon was recombined into pH7FWG2 vector, containing the enhanced green fluorescent protein (eGFP) reporter gene, to generate 35s::*CsWRKY7*-eGFP fusion construct. Then, the recombinant plasmid (35s::*CsWRKY7*-eGFP) and the control (35s::GFP) were transformed into *Agrobacterium tumefaciens EHA105* by freeze–thaw approach [51]. The confirmed bacteria were grown in yeast extract peptone (YEP) medium (pH = 7.4) at 28 °C until optical density at *λ* = 600 nm (OD_600_) to 1.0. This medium added with bacteria was centrifuged at 4000 rpm for 10 min. the sediment was resuspended with suspension buffer containing 10 mM MgCl_2_, 10 mM MES, and 100 μM acetosyringone (AS), with OD_600_ adjusted to 0.4. The suspension was infiltrated into well-developed *N. benthamiana* leaves. After infiltration, the tobacco plant was cultured in darkness for 48 h at 24 °C and then put in light for half an hour. The GFP signal was observed by a confocal microscopy. Additionally, cellular localization of the CsWRKY7-eGFP proteins in lateral roots of transgenic Arabidopsis plants was also observed by confocal microscopy.

### 4.5. Generation of CsWRKY7 Transgenic Plants

Gene-specific primers 5′-CACCATGGCGGTCGAGCTAG-3′ (Forward) and 5′-AGAAGACTCTAAAATGAGACCAGA-3′ (Reverse) were used to clone the open reading frame (ORF) of *CsWRKY7*. The cloned products were inserted into pENTR/D-TOPO vector (Invitrogen, Carlsbad, CA, USA) and sequenced, then the right directional sequence was inserted into pH7FWG2 vector with LR clonase II enzyme mix (Invitrogen) according to the manufacturer’s instructions. The binary vector was transferred into *Agrobacterium tumefaciens strain GV3101* by freeze–thaw method [51], and then this strain was introduced into Arabidopsis by floral dip approach [52]. T4 homozygous seeds were used for the experiments.

### 4.6. Phenotypic Analysis of Transgenic Arabidopsis

For the germination assay, the seeds of WT and homozygous 35S::*CsWRKY7* transgenic lines were germinated on 1/2 Murashige and Skoog (MS) medium containing different concentrations of NaCl (0, 100, 150, 200 mM), mannitol (0, 100, 200 mM), ABA (0, 0.3, 1, 10 μM), and PEG (0, 15%) solution. After 48-h vernalization, seedlings were placed vertically in climate incubator, and germination rate was counted on the fourth day. The germination was defined when the roots break the seed coat by 1 mm. Photographs of the plates with 150 mM NaCl, 200 mM mannitol, 0.3 μM ABA, and 15% PEG were taken on the 7th day. Each plate consisted of WT and three transgenic lines with 50 seeds per line in 9 cm diameter plate. In root length assay, the seeds of WT and homozygous 35S::*CsWRKY7* transgenic lines were sown on 1/2 MS medium. After 48-h vernalization, the plates were placed vertically in climate incubator for 4 d. And then seedlings of similar size were transferred to different stress medium containing 150 mM NaCl, 200 mM Mannitol, 0.3 μM ABA, and 15% PEG, respectively. The root length was measured by ruler after 10-day growth. Arabidopsis seedlings were grown on 1/2 MS medium for 7–10 d, and then transplanted to sterilized soil in a growth chamber for the phenotype observation (16 h day/8 h night at 22/20 °C).

### 4.7. Quantitative Real-Time PCR Analysis in Transgenic Arabidopsis

To investigate the mechanism by which *CsWRKY7* delayed flowering, the expression levels of flowering-related genes in transgenic Arabidopsis lines and wild type were calculated by the 2^−ΔCt^ or 2^−ΔΔCt^ with the expression level of *Actin-2* gene (Gene Locus: At3g18780) as the reference control [49,50]. The primer sequence used for qRT-PCR are listed in Supplementary Table S1.

### 4.8. Statistical Analysis

All assays were performed with either three or more repetitive experiments. Student’s *t*-test was performed to determine the significant difference, and standard error were calculated using the Built-in Functions in Excel 2016. Student’s *t*-test, ‘*’, ‘**’, and ‘***’ indicate significant difference at *p* < 0.05, *p* < 0.01, and *p* < 0.001, respectively.

## Figures and Tables

**Table 1:** Hormone-, light-, and stress-responsive elements in the 1680 bp 5′-flanking sequence of CsWRKY7 TF as predicted by the PlantCARE website.

Types	Site Name	Copy	Sequence	Function
Hormone	TCA-element	2	CCATCTTTTT	salicylic acid responsiveness
	TGA-element	1	AACGAC	auxin-responsive element
Light	Box 4	3	ATTAAT	light responsiveness
	LAMP-element	1	CTTTATCA	light responsive element
	chs-CMA1a	1	TTACTTAA	light responsive element
Stresses	ARE	1	AAACCA	anaerobic induction
	CCAAT-box	1	CAACGG	MYBHv1 binding site
	WBOXHVISO1	2	TGACT	sugar-responsive elements
	WRKY71OS	3	TGAC	A core of TGAC-containing W-box
	WBOXNTERF3	2	TGACY	W-box, wounding
	GT1GMSCAM4	6	GAAAAA	Pathogen- and NaCl-induced expression
	MYB1AT	4	WAACCA	MYB recognition site found in the promoters of *rd22*
	MYB2CONSENSUSAT	2	YAACKG	MYB recognition site found in the promoters of *rd22*
	MYBCORE	4	CNGTTR	responsive to water stress
	CCAATBOX1	4	CCAAT	act cooperatively with HSEs
Development	GTGANTG10	4	GTGA	Late pollen-responsive elements
	POLLEN1LELAT52	8	AGAAA	pollen specific expression

## Supplementary Materials

Supplementary materials can be found at https://www.mdpi.com/1422-0067/20/11/2815/s1. Table S1 Primer sequences used for qRT-PCR.
