# Supplementary material for: Isolation and Characterization of CsWRKY7, a Subgroup IId WRKY Transcription Factor from Camellia sinensis, Linked to Development in Arabidopsis
Source: Int J Mol Sci. 2019 Jun 9;20(11):2815. doi: 10.3390/ijms20112815 (PMC6600228; doi:10.3390/ijms20112815)
Supplement: Supplementary file 1 [file ijms-20-02815-s001.pdf]

Supplemental Table S1 Primer sequences used for qRT-PCR

Genes	Sense primer (5′→3′)	Anti-sense primer (5′→3′)
GAPDH	TTGGCATCGTTGAGGGTCT	CAGTGGGAACACGGAAAGC
CsWRKY7	TCGTGATGGGGAAGAATGGA	CGACGAAGGCTGCTTGCTAT
Actin-2	TGTGCCAATCTACGAGGGTTT	TTTCCCGCTCTGCTGTTGT
SCO1	ATGGTGAGGGGCAAAACTCAG	CCCAATGAACAATTGCGTCTC
LFY	TTGGTGGTTTAGAGGGACTATTCG	GCCGTAAAACCTAACTCCGCTA
AP1	AACCAAGGCCACAATATGCC	CGGGTTCAAGAGTCAGTTCG
CO	GTGATAAGGATGCCAAGGAG	AGTTTAAGCGGAACAACTCTA
FLC	CTCTACAGCTTCTCCTCCGG	TCCCACAAGCTTGCTATCCA
FT	TACGAAAATCCAAGTCCCACTG	AAACTCGCGAGTGTTGAAGTTC
FLM	GATAGAAGCGCTGTTCAAGC	CAGCAACGTATTCTTTCCCAT
